# Analysis and Validation of Cross-Modal Generative Adversarial Network for Sensory Substitution

**DOI:** 10.3390/ijerph18126216

**Published:** 2021-06-08

**Authors:** Mooseop Kim, YunKyung Park, KyeongDeok Moon, Chi Yoon Jeong

**Affiliations:** Human Enhancement & Assistive Technology Research Section, Artificial Intelligence Research Lab., Electronics Telecommunications Research Institute (ETRI), Daejeon 34129, Korea; gomskim@etri.re.kr (M.K.); parkyk@etri.re.kr (Y.P.); kdmoon@etri.re.kr (K.M.)

**Keywords:** sensory substitution, auditory sensitivity, cross-modal perception, generative adversarial network, visual perception

## Abstract

Visual-auditory sensory substitution has demonstrated great potential to help visually impaired and blind groups to recognize objects and to perform basic navigational tasks. However, the high latency between visual information acquisition and auditory transduction may contribute to the lack of the successful adoption of such aid technologies in the blind community; thus far, substitution methods have remained only laboratory-scale research or pilot demonstrations. This high latency for data conversion leads to challenges in perceiving fast-moving objects or rapid environmental changes. To reduce this latency, prior analysis of auditory sensitivity is necessary. However, existing auditory sensitivity analyses are subjective because they were conducted using human behavioral analysis. Therefore, in this study, we propose a cross-modal generative adversarial network-based evaluation method to find an optimal auditory sensitivity to reduce transmission latency in visual-auditory sensory substitution, which is related to the perception of visual information. We further conducted a human-based assessment to evaluate the effectiveness of the proposed model-based analysis in human behavioral experiments. We conducted experiments with three participant groups, including sighted users (SU), congenitally blind (CB) and late-blind (LB) individuals. Experimental results from the proposed model showed that the temporal length of the auditory signal for sensory substitution could be reduced by 50%. This result indicates the possibility of improving the performance of the conventional vOICe method by up to two times. We confirmed that our experimental results are consistent with human assessment through behavioral experiments. Analyzing auditory sensitivity with deep learning models has the potential to improve the efficiency of sensory substitution.

## 1. Introduction

It is a widespread assumption that the visually impaired have superior sensory abilities in other sensory modalities. To verify this assumption, many studies have been conducted to show that visually impaired people have superior auditory sensitivity compared with sighted persons [1]. However, current research has shown that certain auditory abilities are higher in the blind than in sighted controls, such as echo processing and distance discrimination [2,3], while for other abilities such as vertical localization, blindness leads to a deficit [4]. Another related issue is the lack of a systematic and objective methods for the quantification of sensory sensitivity. Although it is not clear whether visual impairment is associated with increased auditory performance, the available evidence demonstrates that visual-auditory sensory substitution has a great potential to compensate for vision loss using audio stimuli [5,6,7]. Various attempts have been made to develop strategies to effectively convey visual information to the blind with auditory stimuli [8,9,10,11,12]. In addition, many studies on visual-auditory sensory substitution have demonstrated the possibility of localizing [13,14] and recognizing objects [15,16], extracting depth and distance [12,17,18], and performing basic navigational tasks [19,20,21].

All of these systems differ in their methods of converting images into sounds. Among these, vOICe [8] is one of the most well-known and widely used methods for psychophysical experiments on visual-auditory sensory substitution. After a proof of concept in the 1990s and with many recent advances, several types of the vOICe system have been developed with various settings, such as the scale of frequency and sound duration for auditory stimuli. Despite the potential benefit of sensory substitution for the visually impaired, vOICe is not widely used in the blind community. It remains one of the most promising approaches, but is currently treated primarily as a research tool [22,23]. Therefore, more efforts should be focused on closing the gap between research under controlled conditions and widespread practical use in the real world. There are several possible reasons why sensory substitution methods have not been adopted by the blind community, such as cost and training issues [24]. Among these high latency between visual information acquisition and auditory output may also contribute to the gap. In general, vOICe converts two-dimensional visual information into a one-dimensional auditory signal at a default rate of one frame of image per second. Although it has been reported that a blind vOICe user experienced smooth movement after training for several months or even years [25], the low conversion rate of the vOICe systems makes it difficult for subjects to perceive fast-moving objects or rapid environmental changes.

The conversion of visual information to auditory signals is relatively limited by the temporal resolution of human hearing. Therefore, it is essential to understand the temporal limitation of auditory sensitivity, which is directly related to the perception of visual information. Traditionally, the evaluation of auditory sensitivity in sensory substitution has been conducted with participants’ feedback. However, human evaluation, in which a number of human participants are involved in a qualitative evaluation, is expensive and time-consuming. Moreover, it is difficult to design an objective method for measuring and analyzing the sensitivity of visual perception owing to significant variations between persons. Because people have different biological and environmental experiences, evaluation results from different participants may vary considerably.

Recently, deep learning has emerged as a breakthrough machine learning technique for various areas in computer science, as well as other disciplines. This advancement of deep learning serves as a motivation to exploit artificial perception models as an alternative to human-based evaluation. A recent study [26] developed two cross-modal perception models with respect to late- and congenitally blind cases. They showed that using a computational model that mimics sensory perception could accelerate optimization evaluation and reduce experimental costs. However, the most important and basic research on auditory sensitivity of visual perception necessary for effective generation of visual-auditory substitution has not been considered in detail.

These factors have motivated our proposal of a new experimental method that is both objective and systematic. The purpose of this work is twofold. First, we objectively evaluate and quantify sensitivity in perception of visual-auditory sensory substitution using a machine learning model. Second, we evaluate the extent to which the results of a model-based evaluation of sensitivity analyses correlated with those obtained through human evaluation.

To achieve these aims, in this study, we employ a deep learning approach to perception with respect to auditory sensitivity in visual-auditory sensory substitution of the visually impaired. Particularly, we investigate the temporal limitation of sound duration of the soundscape using time-compressed audio signals. To this end, we first conducted preliminary experiments to test whether sound compression could be used to verify the transformation in visual perception from audio sounds in both visually impaired and unimpaired subjects. We then exploited a cross-modal perception model, based on deep learning. Although we took inspiration from the cross-modal learning model, the present work differs in that instead of evaluating the encoding schemes of visual-auditory substitution, we used a computational model to estimate the limit of the auditory sensitivity on usability of the vOICe system. In the cross-modal perception for visual-auditory substitution, the generated images represent the perceived visual information from the conveyed audio stimulus. We attempted to measure auditory sensitivity by analyzing the change in perception of images, which are generated in response to the change in the temporal length of the soundscape provided as an audio stimulation. In addition, we compared the proposed method with an evaluation conducted with human participants to investigate whether these two methods were related. In the human evaluation, we aimed to investigate the auditory sensitivities of blind people by means of a design involving three groups of participants, including sighted users (SU), congenitally blind (CB) users, and late-blind (LB) users. Detailed information on group-specific age and gender are provided in Section 4. Compared with previous works, the novelty and key contribution of our work can be summarized in terms of the introduction of a deep learning model to analyze the auditory sensitivity of visual-auditory substitution and of our examination of the correlation between human and model-based evaluations.

If the temporal length of soundscape is reduced, it is expected to become possible to recognize and respond to rapidly moving objects, because surrounding information can be thus transmitted to the blind more quickly. The hypothesis of our study was rooted in the general assumption that the visually impaired have superior auditory abilities compared to sighted persons. If this assumption is true, we predicted that the latency of the conventional sensory substitution device using a soundscape method, which requires a fixed time to convert an image to an audio signal, could be reduced. We also predicted that there would be a difference in the degree to which different participants (SU, CB, LB) perceived the temporally compressed soundscape sound, and that these phenomena could be confirmed experimentally. We conducted two experiments to test our hypothesis.

The remainder of this study is organized as follows: Section 2 reviews the related works. Section 3 presents a detailed description of the proposed methods. The experimental setup and results are presented in Section 4. Finally, conclusions and plans for future work are presented in Section 5.

## 2. Related Work

One important consideration for visual-auditory sensory substitution is whether to convey visual information through sound or speech. With the recent development of artificial intelligence and deep learning, there have been several attempts to convey environmental information to the blind through direct audio semantic description. For example, LookTel [27] detected and identified objects and returned audio feedback of their names in real time. In addition, Microsoft’s Seeing-AI Project [28] attempted to convert visual information into speech to describe people and identify their emotions. In the work of the DEEP-SEE [29] framework, computer vision algorithms and deep convolutional neural networks (CNNs) [30,31] were jointly exploited to detect, track, and recognize objects encountered during navigation in an outdoor environment in real time. The results of object detection were followed by a verbal description and delivered through a bone-conducting headphone. These direct semantic descriptions were easy for users and did not require training, but they had difficulty describing complex environments or shapes of target objects. Another serious factor hindering this approach is that the object and scene categories of the training data are expected to remain limited. In general, training and evaluation of a learning model require large data samples. However, it is expensive and time-consuming to collect and annotate the necessary amount of data.

In contrast, most conventional visual-auditory sensory substitution methods transform visual information into auditory representations using a predefined transformation algorithm. The main difference between conventional methods and direct semantic description is that users need to be trained to be able to understand the relationships between the generated virtual sounds and properties of objects in the image. Although these methods may appear to be difficult to use, they can be more flexible for new environments and unseen objects because they produce sounds based on characteristics directly calculated from the input image. Loomis et al. [11] sought to develop a guidance system to improve navigation without vision. They found that virtual sounds were more efficient for guidance than a semantic description of speech and could be immediately perceived as spatial information. In addition, Väljämae and Kleiner [32] suggested a practical method that converts visual information in a sequential way to generate audio signals corresponding to other dimensions. They found that the brightness of the image could be denoted by loudness of the sound, size with pitch, direction with timbre and so on.

Many prototypes using a method similar to that followed by [32] have already been developed in the field of visual-auditory sensory substitution. As a representative and successful example of this approach, vOICe converts two-dimensional gray tones of a 64 × 64-pixel image into a one-dimensional audio signal through a left-to-right sequential scanning of columns in the image, which is called a soundscape. In this method, the image frame is decomposed into a sequence of columns, and each column is converted into sound during a fraction of the entire period of an image frame. Therefore, from the time-domain perspective, vOICe converts the luminance of each column of the image to a mixture of sinusoidal sounds sequentially from left-to-right across the image. Specifically, pixel brightness in a column is mapped to the amplitude, and the location of the pixel in a column is mapped to a frequency of a sinusoidal audio signal. In addition to using the sequential scanning of columns in images, several studies have focused on improving the auditory pleasantness of the generated sounds. Cronly-Dillon et al. [9] introduced musical notes where frequencies of generated sound increased from bottom to top for each column. To provide both shape and color information, Abboud et al. [10] developed the EyeMusic system, which provided a musical image representation preserving the shape and integrating color information. Despite its simple and intuitive algorithm, these column-wise scanning methods are inconvenient and somewhat obtrusive to the user when perceiving a fast-moving object because of their low conversion rate of a single frame per second.

Other approaches that do not use column-wise scanning but rather extract and convert visual information from whole image frames have also been developed. For example, an earlier method, prosthesis for substitution of vision by audition [33], uses a frequency mapping for visual-auditory substitution. The frequency associated with each pixel increased both from the bottom to the top and from the left to the right sides of the image. To simulate the human visual system, this method used a higher density of auditory pixels in the center of the image. More recently, VIBE [12,34] introduced a virtual retina with two levels of cells consisting of sensors and receptors. Each sensor corresponds to a particular pixel. A receptor has a receptive field, which is activated by a particular area of the image and consists of a set of sensors. These receptive fields are presented simultaneously and each of them can be assigned a specific sound source. Although these methods have the advantage of fast visual-auditory signal conversion, it may be difficult for the user to understand the visual information from the converted audio signal.

Recently, an implicit method for visual-auditory sensory substitution technology, called the autoencoded V2A (AEV2A) model [35], has been proposed. This approach converts visual information of an image into an auditory signal by applying a deep learning algorithm without predefined rules. The AEV2A model builds on the recurrent variational autoencoder (VAE) [36] architecture of a deep recurrent attentive writer [37] to synthesize sound from images and decode the produced sound back as a sequence of drawing operations. Both parts of the encoder and decoder consist of multiple layers of long short-term memory cells. In the case of the AEV2A method, because the deep learning model converts visual information into sound through learning, where the conversion function acts as a black box, it may be difficult for a user to understand the conversion scheme, and thus learning efficiency may be reduced. The authors of [38] applied dynamic time warping to evaluate the consistency of the generated sound against variation in the input image. As a result, no significant correlation was observed between the input image change and the resulting sound obtained from AEV2A. These results show that a small change in the input image resulted in completely different sound generation, indicating the difficulty of learning to use these methods.

All of the above-mentioned efforts to develop visual-auditory sensory substitution methods based on cross-modal perception and various psychological studies [39,40] have discovered a strong correlation in human perception of visual and auditory stimuli. Recently, deep learning has been widely applied and has achieved remarkable success in many fields, and efforts have been made to build an effective cross-modal learning model to simulate the human brain. Ngiam et al. [41] introduced a novel application of deep networks to learn features over multiple modalities. They showed a way to learn the shared representation between modalities and evaluate it, in which a classifier was trained with audio-only data but tested with video-only data and vice-versa. Owens et al. [42] proposed a method to synthesize sound from silent videos of hitting and scratching objects with a drumstick. They used a recurrent neural network to predict sound features from videos and then produced a waveform from these features with an example-based synthesis instead of direct generation. Recently, generative adversarial networks (GANs) [43,44], comprising a pair of neural network models competing with one another, provide an attractive method of learning a generative model for cross-modal generation. The learning objective of GANs is to synthesize realistic data following the distribution of training data. By utilizing this property of the GANs, Chen et al. [45] proposed cross-modal generation models using conditional GANs. They proposed sound-to-image and image-to-sound networks that generate images and sounds, respectively. Instead of using separated models, Hao et al. [46] proposed a unified network for visual-auditory mutual generation using cross-modality cyclic generative adversarial network. More recently, Hu et al. [26] proposed two novel cross-modal perception models for the late and congenitally blind, which aim to generate visual images based on the converted sound. These models were used to evaluate the encoding schemes of vOICe.

## 3. Proposed Method

Research on the evaluation of auditory sensitivity for sensory substitution has been conducted in human behavioral analysis. However, this method relies on the subjective feedback of participants; thus, it cannot be considered an objective method. In this section, we describe a proposed cross-modal GAN to analyze auditory sensitivity in visual-auditory substitution and a behavioral assessment to validate it.

### 3.1. Cross-Modal Gan for Auditory Sensitivity Analysis

As described in previous sections, visual-auditory substitution using a column-wise scan has proven useful and their encoding schemes are considered easy to interpret. However, the low conversion rate prevents the visually impaired from taking advantage of these systems in their daily life because it remains difficult to interpret a dynamically changing environment. A solution to this problem may begin with an accurate and objective analysis of the temporal limitation of the visual information that can be conveyed via soundscape. As a basic approach to solving this problem, we use a deep learning model to analyze the temporal limitation of an encoding scheme that can recognize visual information from the auditory signal. In this section, we describe our approach, which is inspired by the work of [26], to evaluate auditory sensitivity for visual-auditory sensory substitution using a cross-modal perception model.

Our computational model for auditory sensitivity analysis is similar to that proposed by [26], in that it uses the vOICe as an audio embedding for sensory substitution and directly creating visual contents using a cross-modal GAN. However, we extended the model to evaluate how visual perception varies according to the change in the compression ratio of the converted sound. Specifically, the differences in our method include the use of a CNN to perform audio embedding and the use of generated images to evaluate auditory sensitivity. This allows us to evaluate whether there was an effect of change in the temporal limitation of the encoding scheme in visual-auditory sensory substitution.

**Figure 1:**
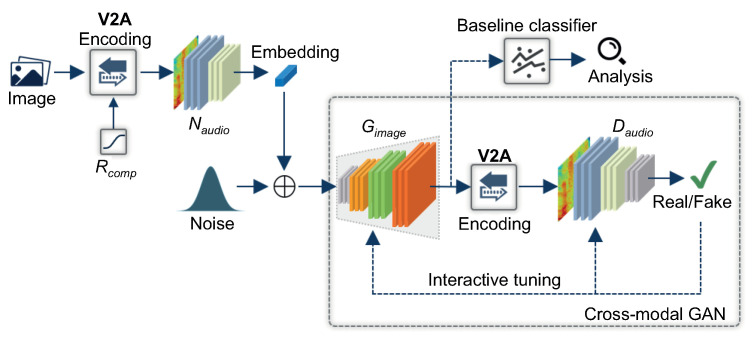
Overall architecture of the proposed computational model to imitate the cross-modal plasticity to perceive visual images through audio signal. A cross-modal generative adversarial network (GAN) learns an image-to-audio mapping using an image generator ($G_{Image}$) and audio discriminator ($D_{Audio}$). V2A encoding represents the vOICe, $R_{comp}$ is the compression ratio, and $N_{Audio}$ is the audio encoder.

Figure 1 shows the architecture of our simulation platform, which consists of networks performing audio embedding and cross-modal GANs. It imitates the plasticity of sensory perception in the human brain by adopting cross-modal translation of visual into auditory information. The primary objective of this section is to investigate the effect of auditory sensitivity on visual-auditory substitution according to the change in the temporal length of the audio signal. Therefore, the input image is first encoded into audio signals. We used the MNIST handwritten digits dataset [47] as the input image for our experiments because it has been used as a benchmark for most previous approaches. For visual-audio encoding, we used the vOICe, and compression ratio was used as a conditional input to control the length of the encoded audio signal. We used 22 kHz as the sampling frequency for audio signals, and the length of the encoded audio signal varied according to the compression ratio.

The encoded audio waveform was then transformed into log-mel spectrogram features as the input to the audio encoder. We used a CNN as our audio encoder to model the abstraction in visual perception from translated audio. It consisted of four convolutional blocks and three fully connected layers. Each convolutional block was composed of a convolutional layer with a kernel size of 3 × 3, a rectified linear unit (ReLU) non-linearity layer, followed by a max-pooling layer with 2 × 2 pooling and 2 × 2 stride window, which effectively down-sampled the output of the prior layer. The last layer was used for classification with softmax loss. For the training of the audio encoder model, we used Adam (Adaptive Moment Estimation) as an optimizer with a batch size of 64 samples per step and a learning rate of 0.0001. We trained the system for 10 epochs. After the network was trained, the last layer was removed, and the feature vector of the second to the last layer with a size of 128 was used as the audio embedding in our cross-modal GAN, which was a variant of the auxiliary classifier GAN (AC-GAN) [48]. In the proposed method, the image generator ($G_{Image}$) takes an audio embedding with a dimension of 128 and a random noise of 100 as a conditional input to generate image samples. The feature maps of the generator enlarged by a factor of four after each upsampling step. The output of the generator was a gray scale image with a size of 28 × 28 pixels. Because the cross-modal GAN handled different modalities, the generated images were not directly input to the discriminator ($D_{Audio}$). Therefore, before input to the discriminator, the system required the vOICe encoding on the generated image and the computation of the log-mel spectrogram on the encoded signal. The discriminator estimated both a probability distribution over sources and a probability distribution over categories. Therefore, the discriminator included two output layers. The first was a single node with a sigmoid activation function to predict real or generated encoded signals. The second predicted the class of the encoded signal using the softmax activation function. An Adam optimizer with a default learning rate (0.001) was used to train the image generator as well as the audio discriminator. To model the perception of visual information from encoded audio signal, the cross-modal GAN model was trained on various numbers of epochs, ranging from 2000 to 10,000, with a batch size of 100 samples. The model began generating images of MNIST digits after 200 epochs.

Note that we employed a cross-modal GAN as only part of the experimental method used in this work to evaluate the perceptual variability according to the change in the compression ratio of the audio signal. To achieve our aim, we introduced an additional classifier to evaluate the variation in the accuracy of generated images from the translated sound of the MNIST dataset. For images, CNNs are known to perform well in various tasks. Therefore, we employed a typical CNN architecture [49] as our baseline classifier model, comprising three alternating pairs of convolutional and pooling layers followed by an additional convolutional layer stacked together. A ReLU was used as an activation function for each of the hidden layer nodes. For model development, the MNIST dataset was divided into training and testing sets using an 80/20 split ratio, and then 20% of the training set was used as a validation set. When classifying the MNIST dataset, the accuracy of the classifier was above 95%; thus, we decided to use this classifier as a baseline classifier model to verify whether the generated images from the vOICe sound were classifiable and belonged to the expected categories.

### 3.2. Behavioral Assessment for Validating Cross-Modal Gan

The purpose of the human evaluation is to expand the experiment by applying the temporal limitation of the auditory sensitivity obtained from the computational model-based assessment. We investigated whether the results of our experiments with human evaluation were consistent with the results of the cross-modal GAN model. We used the MNIST dataset to train a deep learning model for our experiment. However this dataset was limited to 10 categories, and thus was unsuitable for testing sensory substitution, because participants could memorize it. The visually impaired generally prefer to recognize shape first. Therefore, for human evaluation, we used shapes as visual information in order to be faithful to the aim of conversion of images and verification of perception.

To examine the effect of the temporal length of the soundscape that could be used for training, we used three different lengths of sounds (short, normal, and long) for training each group. Reflecting the analytical result that existing studies mainly used soundscapes of two lengths (1 s and 2 s), we used these lengths as normal and long, respectively. In addition, we set the compression ratio for short soundscapes to 50%, considering both the results from the computational model-based analysis, of which more detailed expression will be given below in Section 4.2. Compared to the normal length of soundscapes, this compression rate had a time length of 0.5 s. Because the main purpose of the experiment was to find and verify the limitations of auditory sensitivity for visual-auditory sensory substitution, the experiment was conducted with a focus on the visually impaired. Thirty visually impaired participants (consisting of CB and LB groups, each of 15 participants) were randomly assigned in groups of 5 participants to three groups according to the length of the vOICe encoded soundscape, including Group A (short, 0.5 s), Group B (normal, 1 s), and Group C (long, 2 s). More information on participants for each group will be presented in Section 4. Therefore, visually impaired participants of two groups (CB, LB) trained with different lengths of soundscape for the exploratory experiment. Although the experiment was conducted mainly on the visually impaired, it is necessary to analyze how efficient the experimental result for the compressed soundscape is compared to that of the SU. The simplest approach would have been to include the SU participants in all groups, but this was difficult in terms of practical issues such as cost and difficulty of recruiting sufficient participants during the COVID-19 pandemic. Therefore, we included five SU subjects to conduct the training on normal length sound (Group B) as a reference index for the performance analysis between other groups.

**Figure 2:**
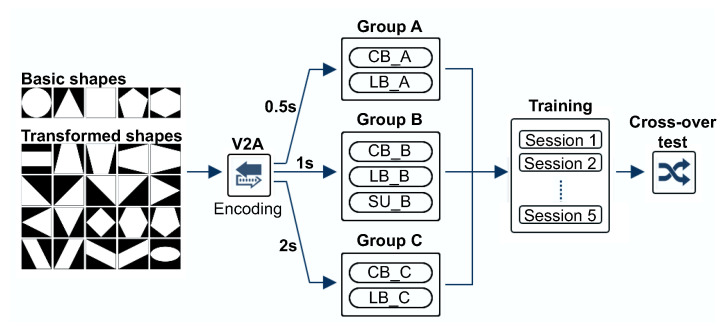
Procedure for experiment on human evaluation. Group A, B, and C refer to groups of participants who trained with sounds encoded in 0.5, 1 and 2 s, respectively, through the vOICe. Each group consists of five participants with congenital blindness (CB) and late blindness (LB). Sighted users (SU) additionally participated in Group B for reference purposes and for comparison of the results of the experiment.

Figure 2 shows the experimental procedure. As visual images, we used 25 simple shapes consisting of 5 basic shapes and 20 variants of these shapes. The auditory stimuli were generated by the vOICe using three types of encoding length, and the generated audio signals were listened to by each participant on Sony MDR7506 headphones. In training sessions, participants were presented with soundscapes, which were converted from images of shapes, and also with associated braille prints to facilitate an understanding of the transferred image shape.

**Figure 3:**
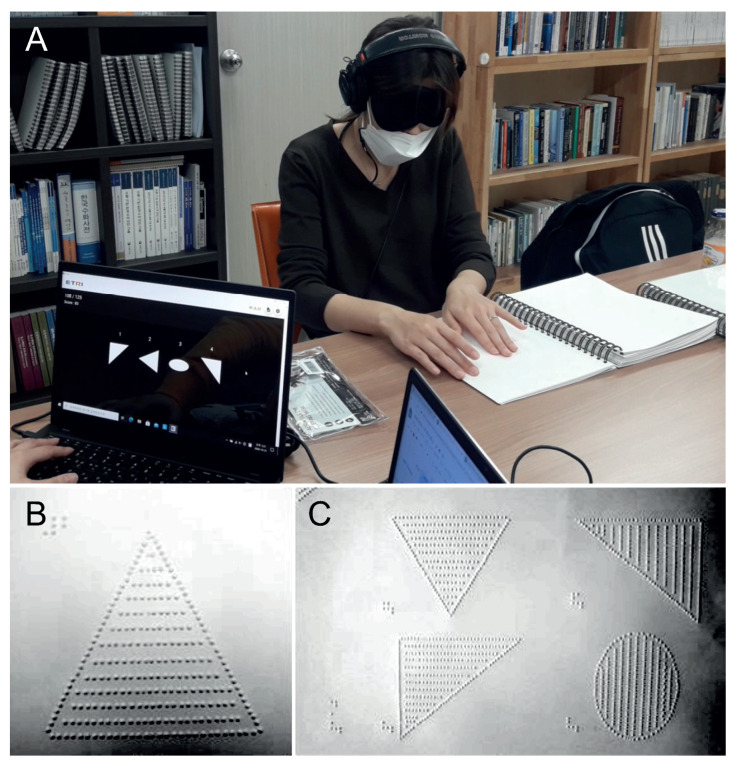
(**A**) The participant wearing a blindfold and listening to the soundscape produced from the shape image displayed on the screen, determining the mappings between shape and sound using the braille print by hand. (**B**) Example of braille print for session training. (**C**) Example of braille print for testing.

White images on a black background were conveyed into soundscapes and each duration of soundscape was fixed to one of the predefined times (0.5, 1 and 2 s), with the frequency on a log-linear scale from 500 Hz to 5000 Hz. At the beginning of each soundscape, we added a 50 ms click sound to inform the participants of the start of the sound. Each image corresponding to the vOICe sound was printed as a braille shape so that the visually impaired could understand the auditory image by exploring it with their hand. Therefore, as shown in Figure 3, all images, braille prints for tactile aid, and associated sound were consistent in dimension.

Because most of the participants of the experiment were visually impaired, the overall procedure of the experiment was conducted in a controlled environment, and the visual images used for each training session were programmed to be selected pseudo-randomly. The participants were given no specific pre-training time to familiarize themselves with the conversion rule of the vOICe, but were fully instructed that the auditory sounds which were to be presented were systematically and closely related to the images. They were asked to use and imagine these relations during their training. The training process consisted of five sessions, each session using 15 image shapes. Throughout the training sessions, every visual image was presented simultaneously with its corresponding sound and braille print. The images used in each session consisted of five basic shape images and ten randomly selected images from the transformed shape images. The 15 images selected in this way were used for training repeatedly in a pseudo-randomized order 10 times for a total of 150 training sets per session. Two small groups of visually impaired (CB, LB) belonging to three groups (Groups A, B, and C) underwent the training with the encoded sound assigned to their group. The participant of CB belonging to Group A can be denoted as CB A, and trained with the sound converted to 0.5 s. All participants of different groups can be identified in this manner.

## 4. Experiment and Results

In this section, we describe the evaluation metrics and analyses of the reliability and validity of the proposed method through experiments. The experiments included a preliminary experiment and a main experiment. Although the authors took initiatives in planning the experiment, defining the problem, presenting a research question, designing the experiment methods, and providing all the materials for the experiment, the participants were recruited and the experiment with the actual test on subjects was performed in cooperation with the Graduate School of Social Welfare, Kangnam University. All the experiments in this study were conducted according to the principles expressed in the Declaration of Helsinki and approved by the Institutional Review Board at the Kangnam University (KNU HR2020010). All the participants provided written and informed consent and received monetary compensation for their involvement. The experimental results are presented and are analyzed below.

### 4.1. Preliminary Experiment

To improve the efficiency of visual-auditory substitution, it is necessary to properly investigate the extent of temporal limitations that can be conveyed through the replacement of audio signals. Unfortunately, this aspect of the behavioral test has been neglected by most previous studies.

#### 4.1.1. Participants

A total of 30 (13 female) participants were recruited into three groups (SU, CB, and LB), and each group consisted of 10 people. Age ranged between 20 and 53 years with a mean age of 33.83 and a standard deviation of 10.52. The demographic information of the participants is summarized in Table 1. As shown in Table 1, the severity grade of all visually impaired participants (S1 to S20) were the most severe level of blindness; the best corrected visual acuity of the better eye is below 0.02 or the radius of visual field is less than 5${}^{\circ }$.

#### 4.1.2. Evaluation of Auditory Sensitivity

**Figure 4:**
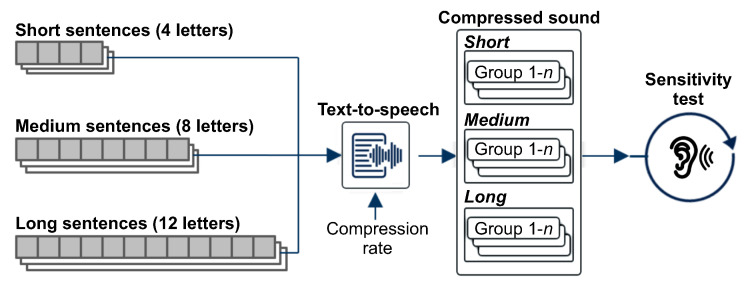
Procedure for the preliminary experiment. Each group of compressed sound corresponded to a predefined compression ratio. In the experiment performed, we used six different compression ratios (*n* = 6).

We conducted an experiment to evaluate the differences between participant groups in auditory sensitivity by changing the compression ratio of the text-to-sound conversion. The purpose of the preliminary experiment was to explore the baseline auditory sensitivity of visual perception for visual-auditory sensory substitution. To examine the temporal limitation of auditory sensitivity, we defined three types of sentences with different lengths in terms of complexity level. According to the authors of [50], the immediate memory span of familiar data such as words and numbers is equal to the amount of data that can be recalled unconsciously for a certain period of time, which is estimated to be approximately 1.5 to 2 s. Based on their findings, we defined the number of letters of sentence for each complexity level as 4, 8, and 12, which correspond to times of 1, 2 and 3 s, respectively. Each complexity level consisted of 10 sentences. Figure 4 shows the procedure for the preliminary experiment.

To obtain more detailed information on auditory sensitivity, the experiment was conducted by increasing the compression ratio from 30% to 70%, which increased linearly by 10%. For audio conversion, each sentence was pronounced by a text-to-speech (TTS) function in response to six predefined compression ratios and the Audition software from Adobe. We performed additional manual work to relieve the crushing of the voice, which was caused by the increase in compression ratio. Thereafter, all the converted audio files were recorded. The sensitivity test consisted of three evaluation sessions corresponding to the complexity level. The experiment progressed in order from short sentences (session 1) to long sentences (session 3). In each evaluation session, participants listened to the TTS-produced sentences using PotPlayer, starting from the sound of the highest compression ratio (70%), to reduce the learning effect through repeated listening.

In the case when a participant could not recognize the meaning of a sentence from the compressed sound, the compression ratio decreased, and the experiment continued with the generated sound by adopting the new compression rate. This process of reducing the compression ratio was repeated until the participant recognized the meaning of the sentence from the TTS-produced sound. When the participant recognized clearly the meaning of the sound being played, the compression ratio of that time was recorded as the sensitivity score of the sentence and the experiment was repeated for the next sentence, beginning with the mostly highly compressed sound.

**Figure 5:**
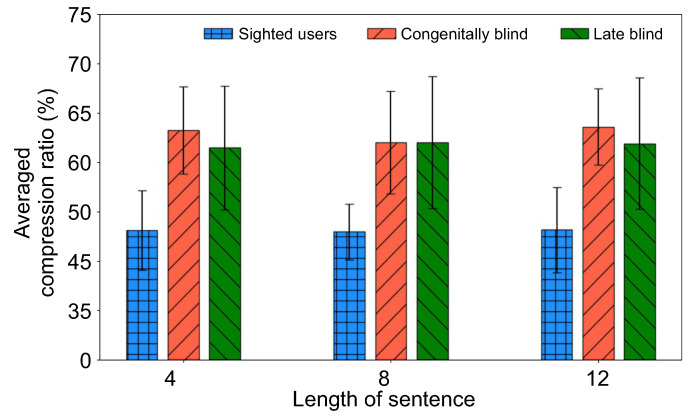
Mean performance obtained by three participant groups as a function of auditory sensitivity. The values of the bar charts indicate the average scores of the tests for each level. Error bars indicate standard errors of the mean.

During the experiment, we recorded the results and compared them with blindfolded sighted participants performing the same experiments. The data collected for this study were statistically processed using the IBM SPSS Statistics 21 tool for empirical statistical analysis. Frequency analysis and descriptive statistics analysis were performed to find out the characteristics of each participant in the experiment. Figure 5 shows the average performance results for three different complexity levels of sentences of the three participant groups categorized as sighted, congenitally blind and late blind. From this figure, it may be observed that there was no significant difference in performance between the two visually impaired groups, but we also found that visually impaired groups had significantly better auditory sensitivity than sighted users in all tasks. More specifically, sighted user groups understood if they heard sentences compressed up to 46% regardless of the length of the sentences. On the other hand, the participants in the visually impaired groups understood the meaning of sentences compressed up to 62–64%. These results show that the visually impaired had higher auditory sensitivity than sighted users.

Among the visually impaired groups, the congenitally blind group showed relatively good performance in the case of the shortest and longest sentences, indicating that subjects were able to extract auditory information with superior sensitivity. This finding confirms the fact that the improvement in auditory performance of visually impaired groups compared to sighted users was indicated by the data recorded. It also suggests that simple text-to-sound conversions can provide a baseline to understand the temporal limitation of auditory sensitivity to visual-auditory sensory substitution.

### 4.2. Evaluation of Auditory Sensitivity Using Cross- Modal Gan

The objective of this section is to investigate the influence of visual perception due to the change in the compression ratio of the vOICe encoding, which is the input for the audio embedding. It is expected that the quality of the generated images from audio embedding worsens as the compression ratio increases. In accordance with this purpose, we first verify the generated images according to the change in the compression ratio of the vOICe encoding. The program for our experiment was implemented using Python 3.5, and the baseline classifier model was implemented using the Keras framework [51,52], based on TensorFlow.

**Figure 6:**
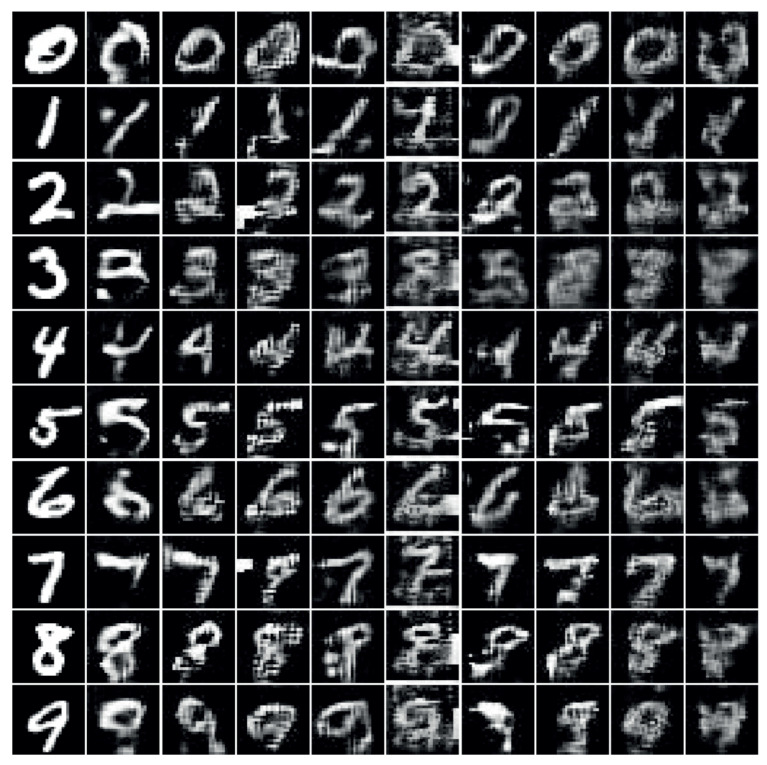
Examples of generated images when different compression rates were used for the vOICe encoding. Each row shows a gradual change of each generated MNIST digit image with the change in compression ratio. The left-most column contains unencoded MNIST digits; the second left-most column contains digits encoded by the vOICe, but not compressed; columns from third left-most to the right contain digits encoded with compression ratios from 10% to 80%, increased by 10%.

Figure 6 shows examples of generated images when MNIST image data were converted to sound while changing the compression ratio of the vOICe encoding. A generated image of a digit corresponds to a class of the translated sound. As may be observed from Figure 6, even when the audio encoding was performed without audio compression, the generated image lead to a loss of image quality compared with the original. Visual examination is the most simple and direct method to observe the change in the generated image by varying the compression rate of the vOICe. However, it was difficult to evaluate the specific extent to which the change in compression rate affected the image quality. Therefore, the development of a method to systematically and efficiently analyze changes in image quality is important. For this purpose, we adopted two widely used metrics for evaluating generated images: the inception score [53,54] and the Fréchet inception distance (FID) [55].

The inception score is a representative metric used to evaluate GANs for image generation using a standard pre-trained inception network [56]. It computes the Kullback–Leibler divergence between the conditional class distribution and the marginal class distribution. To compute the inception score for generated MNIST data, we replaced the pre-trained Inception v3 model with the aforementioned baseline classifier model. The FID metric was also used evaluate generated images in which the inception network was employed. While the inception score simply evaluated the distribution of class probabilities of generated images, the FID compared the values in the embedding layer, which was the second to last layer of the classifier, for real and generated images. Because the inception score was an average of the relative entropy between each prediction and the marginal prediction, a higher inception score represents better quality, while lower FID means that the feature distribution of the generated images was similar to that of real images. To estimate the change in quality of generated images, we measured the inception score and FID after 5000 and 10,000 training iterations were completed.

**Figure 7:**
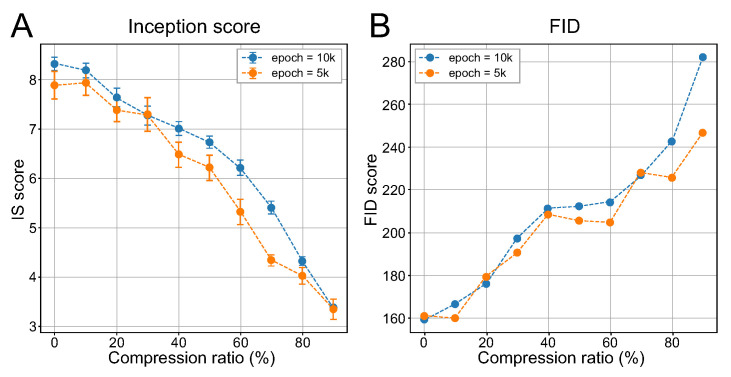
Performance of cross-modal GAN in terms of the (**A**) inception score (IS) and (**B**) Fréchet inception distance (FID) over the change of the compression ratio. The compression ratio for the experiment increased linearly from 10% to 90%.

Figure 7 shows the change in the inception score and FID of the generated images according to the variation in the compression ratio for the vOICe encoding. In our experiment, the compression ratio for audio embedding was linearly increased by 10% with 5000 and 10,000 independently generated samples. As shown in Figure 7, the results for both metrics show similar patterns regardless of training epochs. Our experiment reveals that using the inception score and FID as the evaluation metrics, we can evaluate the effect of the change in compression ratio on the generated images more systematically. From Figure 7, we found that when the compression ratio increased by 60% or more, the quality of the generated images decreased substantially, which was difficult to determine by visual examination.

Based on these results, we further investigated the effect of the variation of the compression ratio on the sensitivity for visual perception. As stated in Section 1, the generated image from cross-modal GAN represents the perceived visual information. Therefore, the generated images could be used as the test data for the baseline classifier model to explore whether there was a change in the accuracy of the baseline model according to the compression ratio variations. Through this method, we could indirectly measure the upper limitation of auditory sensitivity for the perception of visual information transmitted as an audio signal.

**Figure 8:**
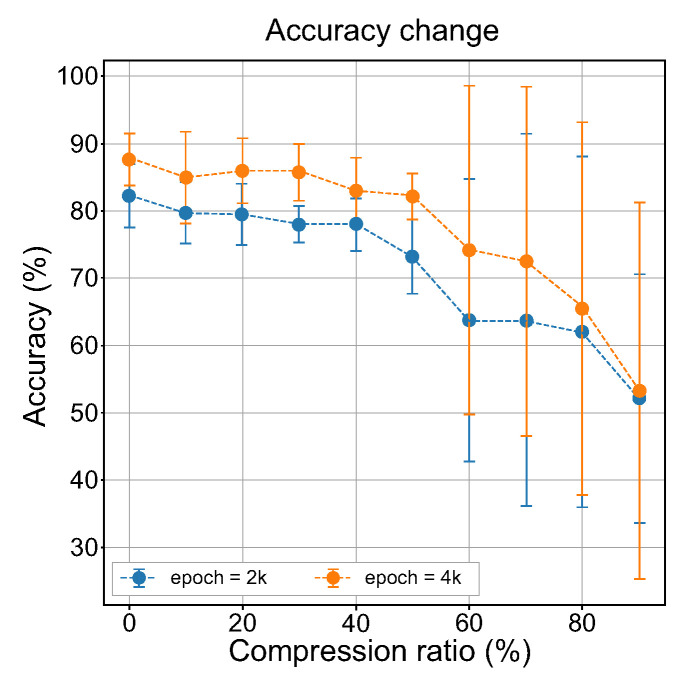
Mean performance change in accuracy of the baseline classifier when the compression ratio for audio embedding increased linearly from 10% to 90%.

To verify our findings from the measurement of the inception score and FID, we repeated the experiment 10 times. Figure 8 shows the averaged measurement results using artificially generated images for the change in accuracy of the baseline classifier that takes place when the audio embedding data are compressed. Because the main purpose of our experiment was not to generate images of good quality using a cross-modal GAN but to observe changes in the perception of visual information, we did not use high numbers of epochs in the experiment.

Interestingly, Figure 8 shows results that exactly match the expected changes in the accuracy of the classifier based on the analysis results of the inception scores and the FID (Figure 7). The average accuracy of the baseline classifier over all generated images continuously decreases as the compression ratio increased. However, it was observed that the standard deviation of the accuracies no longer stabilized from a compression ratio of 60%. This means that the perception of visual information from encoded auditory stimuli began to decrease from this point. These experimental results strongly confirm that the proposed method was able to provide an approximate measure of auditory sensitivity for visual perception without human evaluation.

### 4.3. Main Experiment

From a practical point of view, it is of interest to test and examine whether the computation model-based evaluation of auditory sensitivity for visual perception is consistent with the results of human evaluation. In this section, we compare our computational model-based methods with the human evaluation to investigate whether the results of these two methods showed similar characteristics.

#### 4.3.1. Participants

For the human evaluation experiment, we recruited 35 volunteers (15 women) from 20 to 53 years of age with a mean age of 32.4 and a standard deviation of 9.36. The severity grade of all visually impaired participants (S1 to S30) were the most severe level of blindness. The demographic information of the participants is summarized in Table 2.

During the experimental procedure, we tried to ensure that all participants conducted the experiment in equivalent environments. In particular, as described in Table 2, some participants had remaining weak form vision or had light perception. Therefore, all participants (excluding the participants who were completely blind) were asked to wear blindfolds to ensure the experimental consistency across participant groups.

#### 4.3.2. Evaluation of Auditory Sensitivity Using Behavioral Assessment

As shown in Figure 2, the experiment consisted of five training sessions and one testing session conducted with an interval of a minimum of two to a maximum of four days, each of which took place on a separate day. Each training session consisted of learning and evaluation phases. After each training session, a total of 25 images were repeatedly used times in pseudo-randomized order for a total of 125 test examples per session to evaluate the learning effectiveness of the participants. During the evaluation phase of each session and testing session, each participant was presented with a soundscape, which was played at pseudo-random (each played one time), and four shapes printed in braille (the upper left of each figure was numbered in braille). The participant was then asked to check the shape by exploring with their hand and then asked to choose the correct image of the sound. The training time for each session and the answer to the evaluation for each session were recorded, and no feedback was given to the subject during the session evaluation. Because participants were not instructed to answer as quickly as possible and experiments were conducted without pre-training, the response time in the evaluation was not considered.

**Figure 9:**
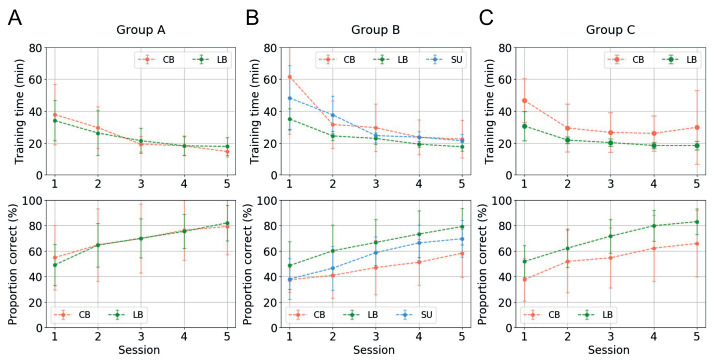
Mean performance and change in the training time (the upper row of the figure) and average proportion of correct answers (the lower row of the figure) obtained by three participant groups in training sessions. Both analyses were plotted against average behavioral score change across subject groups. (**A**) Group A, trained with short (0.5 s) soundscapes; (**B**) Group B, trained with normal (1 s) soundscapes; and (**C**) Group C, trained with long (2 s) soundscapes.

Behavioral performance was measured by calculating the proportion of correct answers on each test with a chance level of 0.25. All analyses of behavioral performance were performed with the average scores of tests of participants and groups. Figure 9 shows the change in average training time and the proportion of correct answers for each training session.

The upper row of Figure 9 shows the change in the average training time taken by the participants of each group to train in a single session during the training process, which consisted of a total of five sessions. In addition, the lower row of Figure 9 shows the average value of each group by observing the accuracy of the shape information transmitted as a soundscape from the four shapes printed in braille in the evaluation, which was conducted after the learning phase of each training session. As shown in Figure 9, the training time required by all participants to learn in each session decreased as learning progressed regardless of the length of the encoded soundscape. Although there were differences in the rates of change in the training times depending on the individual characteristics of the participants, the overall training time decreased significantly. In contrast, the overall degree to which the participants of each group perceived the shapes gradually increased as the training progressed. Our results showed that the LB subjects showed the highest improvement in performance as the training process progressed. The better performance of LB subjects during the training procedure may be the result of their prior experience with shapes or due to the presence of visual imagery.

In our experiment, there was no explanation or prior training of the participants to help them understand the visual-auditory conversion method before proceeding with the training. Therefore, we expected that the change in training time or in visual perception from auditory signals may not appear, or if it appeared, the change would appear very gradually, and that it would be difficult to find meaningful results. However, our experimental results revealed that the process of explaining or pre-training participants was not a prerequisite for sensory substitution. In particular, unlike previous works, an interesting characteristic found in our experiment was that the participants of Group A, who were trained with the encoded sound compressed to half of the normal sound, showed the same change trend as the other groups. This result suggests the possibility that the conversion ratio for visual-auditory substitution of conventional methods, which is the major limitation of the existing vOICe, could be doubled. Moreover, this result also shows that the results of model-based sensitivity analysis and the experimental results of human evaluation are quite consistent.

Following the completion of the training sessions, the testing session was conducted. The testing session was conducted as a crossover test to verify whether all participants could recognize the shape even when they listened to the two lengths of sounds from the other groups that they did not hear in the training sessions. In this test, we expected that we could find important clues regarding the training procedure or protocol for sensory substitution.

**Figure 10:**
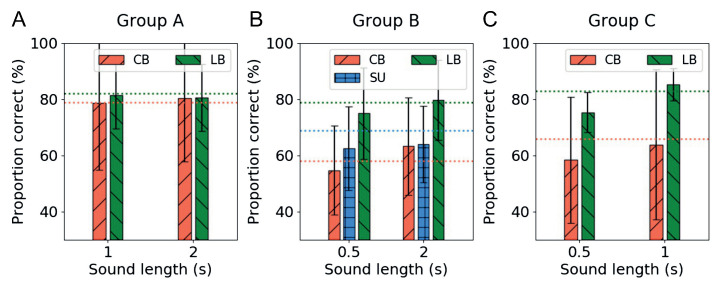
The recognition performance of the crossover test with different length of sounds obtained from three groups of participants. (**A**) Group A, trained with short (0.5 s) soundscapes; (**B**) Group B, trained with normal (1 s) soundscapes; and (**C**) Group C, trained with long (2 s) soundscapes. The x-axis of each figure represents the temporal length of the soundscape used for training the other groups. The dotted lines in the figures represent the mean performance of each group after five training sessions.

Figure 10 shows the averaged performance of each group on the crossover test. Regardless of the compression ratio of the training sound, the performance in all tasks was above chance level. As expected with the performance improvement of the LB participants in the training sessions, they showed a consistent and high performance regardless of the temporal length of the sound in all groups. Group A participants, who trained with the highest compressed soundscape, showed high recognition scores regardless of the temporal length of sound (Figure 10). In contrast, the CB subjects in Group C, who trained with the longest encoded soundscape, had difficulty recognizing information from the given short audio signal. This phenomenon is more clearly explained from the performance results of the CB subjects in Group B, who trained with normal temporal length of sound and had even lower performance than SU subjects when they heard more compressed sound. From this result, we can conclude that in the case of sensory substitution, it is more efficient to use short encoded soundscapes for the learning process of visual-auditory substitution, if possible.

### 4.4. Discussion

Previous studies have proven the technical feasibility of visual-auditory sensory substitution for visually impaired individuals; however, such methods have remained at the level of laboratory research projects or pilot demonstrations without successful adoption in the blind community because of unsolved practical problems. Therefore, it is necessary to find an efficient approach to improve the efficiency and usability of the existing methods. We were interested in examining whether model-based evaluation could be an alternative to human-based behavioral assessment for sensory substitution. In addition, our main question of interest was whether a deep learning model could be used as an objective method to analyze auditory sensitivity in visual-auditory substitution and whether this model had consistent relationship with human-based behavioral assessment.

Our first finding in the preliminary experiment was that the visually impaired generally had superior sensitivity of the auditory sense than sighted users. In general, it is believed that visually impaired people are more sensitive to sound than normal-sighted people; however, many studies have established that visually impaired people are more sensitive to sound than sighted controls for specific tasks. Therefore, we started our work by examining this basic question. Although apparently trivial, the verification of this hypothesis is essential for the efficient development of visual-auditory sensory substitution. Based on the results from our preliminary experiment, we experimentally confirmed that the general assumption about auditory sensitivity of the visually impaired was correct. However, this result remains limited as being found in a preliminary or exploratory experiment, because our experiment was not conducted with a sufficient number of participants.

Our most important finding in the examination is that the auditory sensitivity analysis using cross-modal GAN was consistent with that of human behavioral experiments. From a technical point of view, the most difficult and time-consuming task for sensory substitution is conducting continuous experiments with a large number of participants and analyzing their feedback, because most research in this field has generally been clinically and practically conducted. Therefore, this process requires persistence and patience throughout the entire process from the recruitment of participants to the experiment. Furthermore, it is difficult to obtain consistent results, because they depend on the individual characteristics of participants and the experimental environment. In this study, we used a cross-modal GAN model analyzing the auditory sensitivity of the visually impaired to test whether it was consistent with human evaluation. As a result of the COVID-19 pandemic, it was difficult to recruit enough participants who could complete the experiments within a strict time period. Therefore, it was somewhat difficult to suggest a direct correlation between the model-based analysis and human assessment. Nevertheless, it was revealed that the results of our preliminary experiment and the experiment in which the auditory sensitivity obtained from the model was applied to the actual vOICe encoding were substantially consistent.

The final finding of this study is related to pre-training. According to an analysis in previous research [9,10,11,12], it is important to determine the extent of pre-training necessary in the field of visual-auditory sensory substitution. Our experiment revealed that even if training was conducted without any pre-training, participants were able to recognize visual information to an extent as training progressed if it was fully explained to them that auditory sounds and images were systematically and closely related. It was shown that the training time of the participants decreased significantly as the experiment proceeded, and the perception of visual information increased. The findings stemming from our experiment could be used to provide a guide for future research directions in visual-auditory sensory substitution extending beyond the scope of the current findings.

Generalization remains an important concern. The training of visual-auditory sensory substitution in this study was provided under a controlled experimental setting. Because we used only two-dimensional simple shapes in our experiment, the findings based on this approach may not be extensible to general objects in a more natural environment containing more complex lines and shapes. Therefore, there are various options for future research in terms of developing systematic training methods and procedures to maximize the effectiveness of learning together with the development of model-based efficient encoding methods.

According to the results of our experiment analyzing auditory sensitivity analysis using a cross-modal GAN, the shortest length that could be encoded with vOICe was 0.5 s. We conducted our experiment based on this result. However, we did not conduct further experiment as to whether participants were able to perceive visual information even with a soundscape signal encoded in a shorter temporal length. This could be further extended to a question of whether the shorter latency between visual information acquisition and auditory transduction actually could improve the performance of blind users to perceive a fast-moving object. Therefore, this issue could serve as a future avenue of research that could ultimately lead to the development of more practical aid systems.

Another important application of visual-auditory sensory substitution is the perception of depth and distance and the performance of navigational tasks, as is reviewed in Section 1. Related to this area, it would also be interesting to examine whether the same approach can be applied to analyze the perception of depth information from a visual-auditory substituted signal using a similar deep learning model.

Several existing sensory substitution devices have been developed that convert visual information into an auditory or a haptic signal using a predetermined transformation algorithm [13,57] or other devices that function on the basis of an echolocation principle rather than visual-auditory conversion [58,59]. Because they use different methodologies, the converted sound from visual information are qualitatively different. Moreover, a direct comparison between these methods has never been made, the effect and differences of visual perception due to the differences in substitution method remains unknown. The model-based analysis used in this study is expected to demonstrate the possibility of further exploration of the perception of visual information for these various applications, thus, which could thus serve as topics for future investigation.

Although our experiment using model-based analysis focused on analyzing auditory sensitivity for visual perception, this approach could be extended beyond sensory substitution to evaluate a more systematic understanding of human perception in wider domain areas such as augmented performance [60,61], and human augmentation [62].

## 5. Conclusions

In this study, we have proposed a method for analyzing auditory sensitivity based on cross-modal GAN as a basic approach for the efficient development of visual-auditory sensory substitution. To the best of our knowledge, this is the first effort to analyze auditory sensitivity by applying a deep learning model for sensory substitution and to examine the correlation with human evaluation. The results of the computation model-based analysis show that the auditory sensitivity for visual perception was maintained at up to 60% at the compression rate of the vOICe soundscape.

Before verifying the results of the model-based analysis, we first conducted a preliminary experiment to explore the baseline auditory sensitivity of visual perception. The results of the experiment showed that the visually impaired had up to a 50% higher auditory sensitivity than sighted users. In addition, we expanded our experiment to verify whether participants perceived visual information from auditory stimuli by applying the compression ratio determined by utilizing the results of model-based analysis to the vOICe encoding. As a result of our experiment, by applying the analysis result of auditory sensitivity using a deep learning model, we were able to reduce the temporal length of the auditory signal for vOICe by 50%. This means that we were able to reduce the latency time for sensory substitution by half that, resulting in a performance improvement of 200%. We also demonstrated that we were able to objectively analyze auditory sensitivity using a deep learning model and the overall process of applying the results of model-based analysis to the encoding of sensory substitution was reasonable, consistent overall and sufficiently feasible.

In conclusion, in this study, we have analyzed auditory sensitivity using a deep learning model as a starting point to improve the efficiency of sensory substitution, and evaluated the correlations when the results of the computational model-based analysis were applied to human assessment. While the present work highlights the potential possibility of model-based evaluation for sensory substitution, further research is required to generalize the results of our experiment. Therefore, in our future work, we will aim to answer the fundamental questions that can be asked about visual-auditory sensory substitution, such as, “What kind of visual information should be used for effective learning of sensory substitution?”, “In what order should training proceed?”, “What is the appropriate time for each session of training?”, “How many sessions are required for training?”, and “What is an appropriate time interval between training sessions?” A more comprehensive understanding of these questions would allow us to design a more efficient visual-auditory substitution system, and to develop systematic training protocols for sensory substitution.

## Figures and Tables

**Table 1:** Demographic information of participants for preliminary experiment.

Participants	Assigned Group	Age (Years)	Gender	Time of Sight Loss	Visual Acuity
S1	CB	28	M	1	Light perception
S2	CB	53	M	0	No light perception
S3	CB	20	M	0	Light localization
S4	CB	24	M	0	Light localization
S5	CB	32	F	0	Light perception
S6	CB	31	F	0	Light perception
S7	CB	49	F	3	No light perception
S8	CB	46	M	3	No light perception
S9	CB	41	F	0	No light perception
S10	CB	33	M	0	No light perception
S11	LB	31	M	9	No light perception
S12	LB	36	M	29	No light perception
S13	LB	21	F	11	Form detection
S14	LB	22	M	5	Form Identification
S15	LB	38	M	26	Light localization
S16	LB	44	F	7	Form identification
S17	LB	30	F	15	No light perception
S18	LB	37	F	6	Form detection
S19	LB	24	M	12	No light perception
S20	LB	23	F	13	No light perception
S21	SU	24	M		
S22	SU	46	M		
S23	SU	21	M		
S24	SU	20	F		
S25	SU	46	F		
S26	SU	30	F		
S27	SU	22	M		
S28	SU	39	M		
S29	SU	48	F		
S30	SU	29	F		

**Table 2:** Demographic information of participants for main experiment.

Participants	Exp. Group	Sound Length (s)	Age (Years)	Gender	Time of Sight Loss	Visual Acuity
S1	CB	2	33	M	0	No Light perception
S2	CB	2	32	F	0	Light perception
S3	CB	2	46	M	3	No Light perception
S4	CB	2	49	F	3	No Light perception
S5	CB	2	43	M	3	No Light perception
S6	CB	1	20	M	0	Form detection
S7	CB	1	38	M	0	No light perception
S8	CB	1	53	M	0	No light perception
S9	CB	1	41	F	0	No light perception
S10	CB	1	33	F	1	No light perception
S11	CB	0.5	24	M	0	Light perception
S12	CB	0.5	33	M	0	No light perception
S13	CB	0.5	31	F	0	Light perception
S14	CB	0.5	39	F	0	No light perception
S15	CB	0.5	40	M	2	No light perception
S16	LB	2	21	F	11	Form detection
S17	LB	2	37	F	6	Light perception
S18	LB	2	24	M	20	Light perception
S19	LB	2	44	F	7	Form identification
S20	LB	2	21	M	12	Form identification
S21	LB	1	22	M	5	Form identification
S22	LB	1	38	M	26	Light perception
S23	LB	1	41	M	14	No light perception
S24	LB	1	43	M	14	No light perception
S25	LB	1	30	F	15	No light perception
S26	LB	0.5	23	F	13	No light perception
S27	LB	0.5	36	M	29	No light perception
S28	LB	0.5	31	M	9	No light perception
S29	LB	0.5	35	F	20	Light perception
S30	LB	0.5	33	M	16	Form detection
S31	SU	1	24	M		
S32	SU	1	21	M		
S33	SU	1	20	F		
S34	SU	1	22	M		
S35	SU	1	33	F		

## Data Availability

Data sharing not applicable.
